# Anterior compartment pressure measurement in closed fractures of leg

**DOI:** 10.4103/0019-5413.40261

**Published:** 2008

**Authors:** KC Saikia, TD Bhattacharya, V Agarwala

**Affiliations:** Department of Orthopedics, Guwahati Medical College and Hospital, Guwahati - 32, Assam, India

**Keywords:** Anterior compartment, differential pressure, intracompartmental pressure

## Abstract

**Background:**

Compartment syndrome is a potentially devastating condition. Increased intracompartmental pressure has been incriminated as the primary pathogenic factor in compartment syndrome. The purpose of this prospective study was to monitor the anterior compartmental pressure and differential pressure to minimize the incidence of acute compartment syndrome.

**Materials and Methods:**

Seventy-five consecutive cases of closed fractures of leg presenting within six hours of injury were taken for measurement of anterior compartment pressure at the level of fracture and at 5 cm and 10 cm away from the fracture site, using the Whitesides' infusion technique. A differential pressure of less than 30 mm Hg was taken as the criterion for diagnosis of compartment syndrome.

**Results:**

Two patients (2.67%) developed acute compartment syndrome. The mean anterior compartment pressures were highest at the level of the fracture and went on decreasing as we went away from the fracture site, which was found to be statistically significant (*P* < 0.001).

**Conclusion:**

Compartment pressure measurement is the most reliable and objective method for early diagnosis of compartment syndrome. Whitesides' infusion technique is a relatively easy and inexpensive method to come to a diagnosis of compartment syndrome in a developing country like India. Differential pressure is more reliable than absolute pressure in predicting the development of an impending compartment syndrome.

## INTRODUCTION

Compartment syndrome can be a life or limb-threatening emergency. Early diagnosis is important for the prevention of disability. Approximately 40% of all compartment syndromes occur after fractures of the tibial shaft.^1^ The classical clinical features of five Ps (pain, pallor, paralysis, paraesthesia, pulselessness) cannot be always relied upon for early diagnosis of a developing acute compartment syndrome.^2^,^3^ These features are not apparent in most of the patients unless some permanent damage has occurred. Also, the clinical features are subjective in nature. Intracompartmental pressure measurement is a reliable objective method for early and accurate diagnosis of compartment syndrome.^4^–^10^ Anterior compartment is the most commonly involved compartment of the leg in acute compartment syndrome. Sheridan *et al.*,^11^ Gershuni *et al.*^12^ and McQueen *et al.*,^13^ reported consistent involvement of the anterior compartment in tibial fractures complicated by acute compartment syndrome. As such they stated that monitoring of all four compartments is cumbersome and it seems unlikely that the anterior compartment will not be involved in an acute compartment syndrome. They recommend routine monitoring of anterior compartment and other compartments need to be investigated only if there is clinical suspicion of involvement. Appropriate treatment in the form of decompression of the compartments has to be initiated at the earliest to prevent any permanent disability. The purpose of this prospective study was to monitor and determine the level of the anterior compartment pressure and differential pressure in closed fractures of leg to minimize the incidence of acute compartment syndrome.

## MATERIALS AND METHODS

We studied 75 consecutive patients with closed fractures of leg between July 2004 and December 2006. Only those patients presenting within six hours of injury were included in the study. Patients with compound fractures and who presented after six hours of injury were excluded from the study. Informed consent was taken from each individual. There were 63 male and 12 female patients with the majority in the age group of 21-40 years (65.33%), the mean age being 30.44 years. Only 20% patients had isolated fracture of tibia, the remaining 80% had an associated fracture of the ipsilateral fibula. Right-sided involvement was seen in 46 patients (61.33%). Middle third was fractured in 44 patients (58.67%) and comminution was present in 21 patients (42%). The fractures were classified according to Oestern and Tscherne (1984) classification for closed fractures of tibia into four grades - C0 (n = 5), C1 (n = 47), C2 (n = 19) and C3 (n = 4). This classification is based on the extent of soft tissue abrasions and contusions, the radiological features of the fracture, the presence of closed degloving, the rupture of major blood vessels and the presence of a compartment syndrome.^14^ Distribution of the age, sex and type of fracture are detailed in Table 1.

**Table 1:** Type of fracture within each age group and sexes

Age group (in years)	Number of patients (%)	Sex distribution	Type of fracture*			
			
			C0	C1	C2	C3
01-10	1 (1.33)	M = 1	1	-	-	-
		F = 0	-	-	-	-
11-20	16 (21.33)	M = 15	-	7	7	1
		F = 1	-	1	-	-
21-30	24 (32)	M = 19	2	13	3	1
		F = 5	1	3	1	-
31-40	25 (33.33)	M = 20	-	14	5	1
		F = 5	-	4	1	-
41-50	4 (5.33)	M = 3	1	1	-	1
		F = 1	-	1	-	-
51-60	4 (5.33)	M = 4	-	3	1	-
		F = 0	-	-	-	-
61-70	1 (1.33)	M = 1	-	-	1	-
		F = 0	-	-	-	-
Total	75		5	47	19	4

* - Oestern and Tscherne (1984)^14^ classification for closed fractures of tibia; M - Male, F - Female

All the patients were evaluated for the presence of any associated life-threatening emergency and as such resuscitation was carried out for these patients (n = 9). A careful physical examination was carried out to look for the clinical features of compartment syndrome including pain out of proportion with firmness of the compartment, pain on passive stretching of the involved muscles as well as paralysis, paraesthesia and pulselessness. Proper radiographs of the involved extremities were taken. Anterior compartment pressures of the injured extremities were measured using the Whitesides' infusion technique.^6^

**Figure 1:**
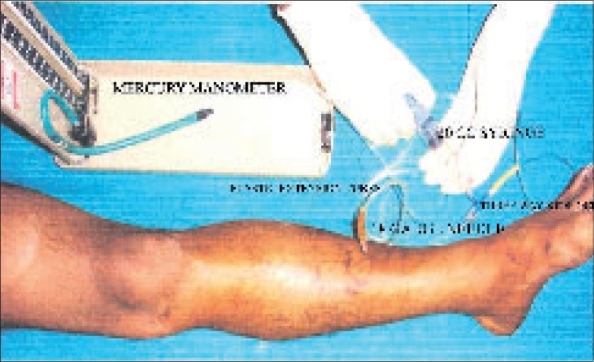
Anterior compartment pressure measurement using Whitesides' technique

Whitesides' technique employs the following materials - i) One mercury manometer, ii) Two plastic intravenous extension tubes, iii) Two 18-gauge needles, iv) One 20-cc syringe, v) one three-way stopcock, vi) One bottle of bacteriostatic normal saline [Figure 1]. The extremity to be measured is cleaned and sterility prepped. Sterile saline is drawn into the 20 ml syringe, which is attached to the three-way stopcock. A single intravenous extension tube is attached to the stopcock and a second 18-gauge needle is attached to its other end. The third unused portion of the stopcock is closed off temporarily. The 18-gauge needle at the end of the extension tube attached to the stopcock is then inserted into the bottle of the saline. Saline is then aspirated without the bubbles into approximately half the length of the extension tube. The three-way stopcock is turned to close off this tube so that the saline is not lost during transfer of the needle. The second extension tube is then connected to the three-way stopcock at its remaining open part and its other end is connected to the manometer. The saline-containing needle is then inserted into the muscle of the extremity to be tested. The stopcock is then turned so that the syringe is opened to both extension tubes, forming a T-connection with a free column of air extending from behind the column of saline into the syringe as well as into the manometer. Pressure is increased in the system gradually by slowly depressing the plunger of the syringe while watching the column of saline. As the plunger is depressed, the saline meniscus will be altered from a convex configuration to a flat configuration, when the air pressure in the system equals the interstitial pressure in the patient's examined tissue. The manometer reading at this time is the tissue pressure in mm Hg. Precautions were taken not to depress the syringe plunger too rapidly or placing the needle into the tendon, as these may give a false high reading. A new needle was used for each measurement in order to assure accuracy.

Pressure measurements were taken at the level of fracture and at 5 cm and 10 cm away from the fracture site proximally and distally. Differential pressures were calculated by subtracting the absolute tissue pressure from the patients' diastolic blood pressure.

Patients with high absolute tissue pressure (>50 mm Hg) were subjected to repeat measurements after one or two hours. The diagnosis of impending compartment syndrome was made when the differential pressure was less than 30 mm Hg.

## RESULTS

**Figure 2:**
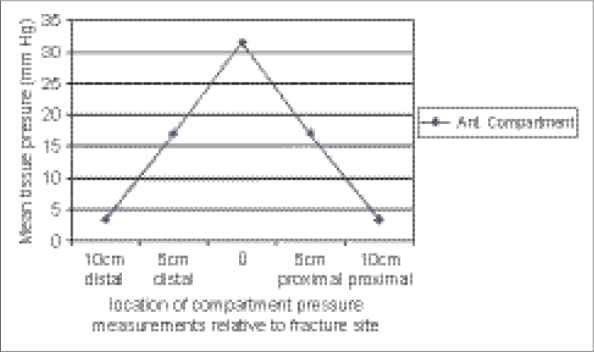
Graph distribution of mean anterior tissue pressure at fracture site, 5 cm and 10 cm proximally and distally

Road traffic accident was the commonest mode of injury and accounted for 62.67% of the cases in our series. Other modes of injury included domestic falls (16%), fall from height (13.33%), sports injury (6.67%) and assault (1.33%). The compartment pressure was found to be highest at the level of the fracture. A pressure gradient was established as we moved away from the fracture site. The mean anterior compartment pressures at the level of fracture and at 5 cm and 10 cm away from the fracture site were found to be 31.47 mm Hg, 17.07 mm Hg and 3.41 mm Hg respectively. The mean difference in pressure at 5 cm from the highest level recorded was 14.4 mm Hg and at 10 cm was 28.05 mm Hg. Table 2 shows pressure distribution at different levels of fracture with mean tissue pressure (mm Hg) in Figure 2. Comparison of the pressures of the anterior compartment measured at different levels of the fracture shows *p*-values of < 0.001, which is statistically significant. Statistical analysis was done using Fisher's exact test.

**Table 2:** Pressure distribution at different levels of fracture

	Number	Minimum	Maximum	Mean	Std. Deviation
ANT O	75	12	62	31.467	9.9693
ANT 5	75	6	36	17.067	7.3325
ANT 10	75	0	14	3.413	4.1168
SBP	75	100	170	122.77	15.8118
DBP	75	60	110	79.306	9.5372
Diff. Pr	75	16	84	47.973	12.9948

Ant 0, Ant5, Ant10 - Anterior compartment pressures at 0 cm, 5 cm, 10 cm away from fracture site respectively; SBP - Systolic blood pressure; DBP - Diastolic blood pressure; Diff. Pr. - Differential pressure

On comparing the mean anterior compartment pressure in different modes of injury, it was found to be more in patients sustaining injuries following road traffic accidents.

Based on the results of the absolute anterior compartment pressures (ACP), differential pressures (DFP) and the presence or absence of clinical features, the patients were divided into four groups.

**Group I:** patients with ACP < 30 mm Hg and DFP > 30 mm Hg. There were 33 patients in this group.

**Group II:** patients with ACP > 30 mm Hg and DFP > 30 mm Hg with no clinical signs of raised pressure. There were 21 patients in this group.

The patients in Group I and Group II were diagnosed as having no compartment syndrome. The fractures in these groups were treated with immobilization in plaster cast or internal fixation as appropriate.

**Group III:** patients with ACP > 30 mm Hg and DFP > 30 mm Hg with clinical signs of raised pressure. There were 19 patients in this Group. All these patients were kept under observation and treated with splitting of the casts and paddings. The limb was elevated to the level of the heart and hyperbaric oxygen inhalation administered. Repeat pressure measurements were performed at every one or two-hourly intervals until the pressure fell down to the safe range.

**Group IV:** patients with ACP > 30 mm Hg and DFP < 30 mm Hg. These patients were diagnosed as having acute compartment syndrome and were treated with four-compartment decompression using the double incision fasciotomy technique. In our series only two out of 75 patients developed acute compartment syndrome (incidence 2.67%). Fasciotomies were performed in both the patients within eight hours of injury and the fractures were stabilized with tibial interlocking nails.

### Case 1

A 26-year-old male riding a motorbike was struck by a truck and sustained closed fracture of right tibia and ipsilateral fibula. The patient had associated exhaust pipe thermal burn over the anterolateral aspect of the leg. The fracture was in the middle third of the leg. Clinically, there was only swelling of the compartment with mild pain at the fracture site. The absolute anterior compartment pressure was 54 mm Hg at the level of the fracture and the differential pressure was 16 mm Hg. A double incision fasciotomy of all the four compartments was carried out within six hours of injury. There was no evidence of muscle necrosis. The fracture was stabilized with interlocking nail. Primary wound closure was not attempted. The wounds were covered with split skin graft after five days. The fracture united in 15 weeks time. The patient did not have any sequelae of acute compartment syndrome at the latest follow-up at 22 months after injury, except for the presence of a scar over the lateral wound.

### Case 2

A 29-year-old male sustained closed fracture involving the middle third of both bones of the right leg following injury by a speeding vehicle. The fracture was non-comminuted. The patient had stretch pain and swelling of the compartment. The absolute anterior compartment pressure was 48 mm Hg and the differential pressure was 22 mm Hg. Four-compartment decompression was performed using double incision fasciotomy within eight hours of injury. The fracture was stabilized with interlocking nail. Medial wound was closed primarily and the lateral wound was covered with a split skin graft after seven days. The fracture united in 18 weeks time. He did not have any neuromuscular deficit at the latest follow-up (16 months) after injury, except for a scar over the lateral wound.

## DISCUSSION

Acute compartment syndrome is an acute surgical emergency.^5^ Early diagnosis and treatment are of the utmost importance in order to avoid long-term disability.^11^ The clinical features cannot always be relied upon for diagnosis. Compartment pressure measurement is the most reliable and objective method for early diagnosis.^4^–^11^ A number of devices are available to measure intracompartmental interstitial pressure like Wick catheter, Simple needle manometry, Slit catheter, Side-porter needle, Fibreoptic transducer. Most of these devices are either expensive or not easily available in developing countries. Wick catheter and slit catheter has the danger of being left behind in the compartment. Whitesides' apparatus is one of the devices used for measurement of tissue pressure. The apparatus is simple and effective and can be assembled with the materials easily available in any hospital ward or emergency room. Moreover, it is inexpensive, safe, reproducible and most importantly ideal for use in peripheral hospitals in a developing nation like ours.^15^,^16^

It is evident from our study that young patients, especially men and those sustaining road traffic accidents are at increased risk of developing compartment syndrome.

The critical level of absolute tissue pressure above which decompression should be performed has always been variably reported as 30 mm Hg,^17^–^19^ 40 mm Hg,^20^ 50 mm Hg.^4^ Whitesides *et al.*, introduced the concept of relative or differential pressure, stating that ischemia begins when pressure rises to within 10 to 30 mm Hg of the diastolic blood pressure.^6^ According to McQueen *et al.*, absolute compartment pressure is an unreliable indication of the need for fasciotomy.^21^ There is now a growing body of evidence to support this view. Setting an absolute pressure ignores the role of the blood pressure in maintaining an adequate blood flow within the compartment. Tissue viability is dependent on adequate perfusion and the blood flow within the microcirculation is dependent on both tissue and venous pressures.^22^

We have also taken the differential pressure of less than 30 mm Hg as the criterion for diagnosis of acute compartment syndrome.^6^,^23^ In a study using this differential pressure for surgical intervention it was demonstrated that many unnecessary fasciotomies were avoided, while patients who had developed an increase in compartmental pressure sufficient to cause obvious tissue compromise as seen at the time of fasciotomy were identified correctly.^20^ Had we used the absolute pressure of more than 30 mm Hg as the threshold for decompression, as recommended by many authors, then 40 out of 75 patients (53.33%) would have had an unnecessary fasciotomy.^16^–^18^ The highest pressure recorded in a patient who did not require fasciotomy was 54 mm Hg, but this was in the presence of a differential pressure of 38 mm Hg. None of the patients in our series had any stigmata of compartment syndrome during a minimum follow-up of six months. This proves that not a single case of compartment syndrome remained undiagnosed on taking the differential pressure of less than 30 mm Hg as the criterion for diagnosing acute compartment syndrome.

Heckmann *et al.*, reported a relationship between compartmental tissue pressures and the distance from the site of the fracture.^23^ Pressure was measured at the fracture site and in 5-cm increments distal and proximal. In their series, 89% of the compartments had the highest pressure measurement at the fracture site; 5% at 5 cm distal and 2% at 5 cm proximal. The mean difference in pressure 5 cm from the highest level recorded was 10 mm Hg. In the present series also, we have found that the peak pressure in a compartment always lies within 5 cm of the fracture site. A pressure gradient is established as we move away from the fracture site, which is statistically significant (*P* < 0.001). Pressure measurements, therefore, should be carried out as close to the fracture site as possible. Our findings are consistent with those of Heckman *et al.*^23^

From our study, we conclude that intracompartmental pressure monitoring is a reliable and objective method for early and accurate diagnosis of compartment syndrome. Early diagnosis can minimize the soft tissue damage and therefore improve the long-term results. We therefore believe that all patients with closed fractures of leg should have routine anterior compartmental monitoring. Whitesides' technique, though not much widely favored, is a safe, inexpensive, easily assembled and reliable method for measurement of intracompartmental pressure in a peripheral setup in developing countries.
